# Association between HIV genotype, viral load and disease progression in a cohort of Thai men who have sex with men with estimated dates of HIV infection

**DOI:** 10.1371/journal.pone.0201386

**Published:** 2018-07-31

**Authors:** Wanna Leelawiwat, Sarika Pattanasin, Anuwat Sriporn, Punneeporn Wasinrapee, Oranuch Kongpechsatit, Famui Mueanpai, Jaray Tongtoyai, Timothy H. Holtz, Marcel E. Curlin

**Affiliations:** 1 Thailand Ministry of Public Health–U.S. Centers for Disease Control and Prevention Collaboration, Nonthaburi, Thailand; 2 Division of HIV/AIDS Prevention, Centers for Disease Control and Prevention, Atlanta, Georgia, United States of America; Instituto de Salud Carlos III, SPAIN

## Abstract

**Background:**

Differences between HIV genotypes may affect HIV disease progression. We examined infecting HIV genotypes and their association with disease progression in a cohort of men who have sex with men with incident HIV infection in Bangkok, Thailand.

**Methods:**

We characterized the viral genotype of 189 new HIV infections among MSM identified between 2006–2014 using hybridization and sequencing. Plasma viral load (PVL) was determined by PCR, and CD4+ T-cell counts were measured by flow cytometry. We used Generalized Estimating Equations to examine factors associated with changes in CD4+ T-cell counts. Factors associated with immunologic failure were analyzed using Cox proportional hazard models.

**Results:**

Among 189 MSM, 84% were infected with CRF01_AE, 11% with recombinant B/CRF01_AE and 5% with subtype B. CD4+ T-cell decline rates were 68, 65, and 46 cells/μL/year for CRF01_AE, recombinants, and subtype B, respectively, and were not significantly different between HIV subtypes. CD4+ T-cell decline rate was significantly associated with baseline PVL and CD4+ T-cell counts (p <0.001). Progression to immunologic failure was associated with baseline CD4+ T-cell ≤ 500 cells/μL (AHR 1.97; 95% CI 1.14–3.40, p = 0.015) and PVL > 50,000 copies/ml (AHR 2.03; 1.14–3.63, p = 0.017). There was no difference in time to immunologic failure between HIV subtypes.

**Conclusion:**

Among HIV-infected Thai MSM, low baseline CD4+ T-cell and high PVL are associated with rapid progression. In this cohort, no significant difference in CD4+ T-cell decline rate or time to immunologic failure was seen between CRF01_AE and other infecting HIV subtypes.

## Introduction

In the absence of antiretroviral therapy (ART), infection with HIV causes a gradual weakening of the host immune system, leading to acquired immune deficiency syndrome (AIDS), and premature death. The time from infection to the development of the clinical diagnosis of AIDS varies between individuals, but more rapid progression has been reported among homosexual men with a median of 7 to 10 years.[1–7]

In contrast, for reasons not yet fully identified, some individuals experience long-term asymptomatic infection.[8–12] Plasma viral load and peripheral CD4+ T-cell count both have utility as indicators of progression of HIV infection towards clinical disease. Prior studies suggest factors influencing the rate of HIV disease progression and include host genetics, gender, age, ethnicity, and viral genetic features such as co-receptor tropism and HIV-1 subtype.[13–20]

The HIV epidemic can be characterized by distinct viral subtypes and circulating recombinant forms loosely segregated by geographic regions and transmission risk groups. The HIV epidemic in Thailand was initially comprised of predominantly HIV subtype B’ infections among people who inject drugs (PWID), with a smaller number of CRF01_AE infections primarily among heterosexual individuals. However, molecular studies conducted since the late 1990s suggest a rising predominance of CRF01_AE and complex recombinant strains in all risk groups.[21–24] HIV viral strains may differ in important respects such as replicative fitness, coreceptor usage, and host cell tropism. Whether circulating HIV subtypes differ clinically in a meaningful way remains unclear. Several groups have investigated the impact of HIV subtype on disease progression but the results have been inconsistent.[25–27]

We previously described a complex and evolving HIV molecular epidemiology among MSM in Bangkok, Thailand.[22] To investigate the association between HIV subtypes and disease progression, we sought to examine differences in markers of disease progression in Thai MSM infected with various HIV strains in common circulation in Thailand. In this study, we determined the estimated rate of CD4+ T-cell decline, the proportion reaching immunological failure, defined as CD4+ T-cell count <200 cells/μl, or initiation of ART, and the estimated time from infection to immunologic failure among MSM infected with HIV subtype B, CRF01_AE, and B/CRF01_AE recombinant strains. We examined the association between baseline clinical characteristics, infecting HIV genotype, and these markers of disease progression.

## Methods

### Ethics approval

The Bangkok Men Who Have Sex with Men Cohort Study (BMCS) protocol was reviewed and approved by the Thailand Ministry of Public Health Ethical Review Committee for Human Subjects Research and by the Institutional Review Board of the U.S. Centers for Disease Control and Prevention. Informed consent was obtained from all participants prior to enrollment in the study.

### Study subjects and detection of incident HIV infection

Between April 2006 and November 2010, 1744 MSM age ≥18 years were enrolled in the BMCS, 1372 with negative anti-HIV antibody screening test results.[28] Study participants were followed every four months to determine HIV-infection status (OraQuick, Orasure Technologies, Bethlehem, PA, USA). Positive oral-fluid antibody results were confirmed using three consecutive rapid HIV antibody blood tests: 1) Determine, (Alere, Tokyo, Japan), 2) DoubleCheck or DoubleCheck Gold (Orgenics, Yavne, Israel), and 3) SD Bioline (Standard Diagnostics, Gyeonggi-do, Korea) or Core (Core Diagnostics, Birmingham, UK). Participants with negative oral-fluid test results were screened for acute HIV infection by fourth generation enzyme-linked immunoassay (EIA, AxSym or Architect HIV 1/2 Ag/Ab Combo, Abbott, Wiesbaden, Germany) and nucleic acid amplification testing (NAAT, Aptima Genprobe, Hologic, San Diego, CA, USA). All HIV-infected study participants were referred for standard HIV treatment at the time of diagnosis and at subsequent follow-up visits.

### CD4^+^ T-cell count and PVL determination

Newly HIV-infected participants provided blood specimens immediately after recognition of HIV infection (“baseline sample”), and every 4 months thereafter. ART use was ascertained at each study visit through verbal interview. CD4+ T-cell count was performed at baseline and every 4 months on whole blood by single platform volumetric flow cytometry (Guava EasyCyte Plus System, Guava Technologies, Inc., Hayward, CA USA). HIV RNA was quantified in plasma collected at baseline using COBAS TaqMan HIV version 1.0 and 1.5 (Roche Molecular Systems, Branchburg, NJ, USA). The lower limits of detection were 47 and 34 copies/mL, respectively. Plasma without HIV RNA detected was recorded as 0 copy/mL.

### HIV genotyping

HIV genotype was determined using multi-region hybridization (MHAbce version 2) and sequencing as previously described.[29] Briefly, HIV RNA was extracted from 200 μL of plasma using the QIAamp Viral RNA Mini Kit (Qiagen, Hilden, Germany). Reverse transcription and PCR amplification for partial HIV genes (*p17*, *gag*, *rt*, *int*, *tat*, *gp120*, *gp41*, *nef*) spanning the HIV genome were performed. HIV Taqman probes specific for subtype B, CRF01_AE and C were used to hybridize PCR amplicons. HIV genotypes were classified by MHAbce according to established criteria. [22] MHAbce results suggestive of dual infection and/or inter-strain recombination were confirmed by gene sequencing; amplicons from all gene regions showing possible dual infections or recombination were used for bulk sequencing (ABI 3130 Capillary sequencer, Applied Biosystems, Foster city, CA, USA). Visual inspection of neighbor-joining phylogenetic trees was used to classify infections as HIV genotype B, CRF01_AE, recombinant B/CRF01_AE or HIV genotype B and CRF01_AE dual infections. Those infected with dual infections and recombinant strains were combined in one group for statistical analyses. All sequence data derived from samples included in this study was submitted to Genbank (accession number MH517658-517678).

### Statistical analyses

Participants with known HIV genotypes and two or more follow-up CD4+ T-cell measurements were included in this analysis. The maximum follow-up duration used for calculations was 80 months. Persons who initiated antiretroviral therapy (ART) were censored at their last pre-ART visit. The estimated date of infection (EDI) was defined as the midpoint between the date of last negative (LN) and first positive (FP) HIV test. Baseline CD4+ T-cell count and PVL were defined as the first available results within 120 days of EDI. Baseline characteristics including age, time from LN to EDI, length of follow-up, and laboratory measures were compared by infecting HIV genotype using the Kruskal-Wallis test. The rate of CD4+ T-cell decline was calculated based on changes of CD4+ T-cell per day as an average of [(CD4^+^_Ti+1_- CD4^+^_Ti_)/ (t _i+1_-t_i_)], where T_i_ was the follow-up visit of the individual (i = 1, 2, 3, …, 17) and t _i_ was the visit date of individual. The median value was reported as the rate of CD4+ T-cell decline per year. A linear Generalized Estimating Equations (GEE) model with an exchangeable correlation matrix was used to examine factors associated with changes of CD4+ T-cell over time. This allowed us to account for correlations of repeated measurements within subjects. Variables with a *p* value ≤ 0.2 in bivariate analysis (adjusted on “visits”) were included in the multivariable analysis.

Survival analysis using the Kaplan-Meier survivor function was used to describe disease progression from EDI to immunologic failure, defined as 1) CD4+ T-cell < 200 cells/μL; or 2) Initiation of ART. Time from EDI to the first instance of either endpoint was calculated. Cox proportional hazard modeling was used to determine predictors of immunologic failure. Variables with a p value ≤ 0.2 in bivariate analysis were included in the multivariable model. Statistical significance was evaluated using a two-sided p value of 5%. All analyses were performed with Stata version 12 (Stata Corp LP, College Station, TX, USA).

## Results

### Study participant characteristics

Between April 2006 and December 2014, 246 MSM were identified as being newly HIV-infected individuals. Of these, 213 returned for blood collection and had HIV genotyping testing, and 189 (89%) could be typed and had 2 or more CD4+ T-cell measurements. The median time from last negative date to EDI was 61 days (interquartile range [IQR] 59–66 days) and median follow-up time from first to last CD4+ T-cell count was 37 months (IQR 6–74 months) with a median of 8 CD4+ T-cell measurements (IQR 2–15 measurements) prior to ART initiation. Among these 189 MSM, 158 (84%) were infected with CRF01_AE, 10 (5%) were infected with genotype B, and 21 (11%) had either recombinant B/CRF01_AE or dual infections. There was no significant difference between MSM infected with different HIV genotypes with respect to: age at HIV conversion, elapse time between last negative and EDI, length of follow up time, baseline CD4+ T-cell count, or baseline VL (Table 1).

**Table 1:** Baseline characteristics of MSM infected with HIV-1 subtype B, CRF01_AE and recombinant B/CRF01_AE, Bangkok Men Who Have Sex with Men Cohort Study, Bangkok, Thailand, 2006–2014.

Characteristics	Infecting HIV-1 subtype				
	Overall	Subtype B	CRF01_AE	Recombinant B/CRF01_AE	p value
Number of infected subjects, n (%)	189	10 (5%)	158 (84%)	21 (11%)	-
Age at HIV-1 conversion, median (range) years	26 (18–51)	27 (21–33)	26 (18–51)	26 (19–34)	0.83
Time from LN to EDI, median (range) days	61 (2–812)	62 (58–78)	61 (2–786)	61 (28–812)	0.70
Length of follow up, median (range) months	37 (6–80)	38 (6–56)	37 (6–80)	37 (6–64)	0.60
Number of CD4 measurement, median (range) times	8 (2–18)	8 (2–13)	9 (2–18)	8 (2–14)	0.83
Baseline CD4, median (range) cells/μL	483 (120–1185)	536 (281–736)	465 (120–1185)	519 (347–819)	0.27
Baseline VL, median (range), copies/mL	89100 (0–4454485)	52200 (0–367000)	98100 (391–4454485)	89100 (5490–1178772)	0.28

### Factors associated with rate of CD4+ T-cell decline

The overall estimated rate of CD4+ T-cell decline was 67 cells/μL per year (IQR: -262 to 109 cells/μL). MSM infected with CRF01_AE had greater CD4+ T-cell decline rate (median 68 cells/μL per year; IQR: -258 to 116 cells/μL) than those infected with recombinant B/CRF01_AE (median 65 cells/μL per year; IQR: -294 to 83 cells/μL) and genotype B (median 46 cells/μL per year; IQR: -263 to 81 cells/μL), but the differences between groups were not statistically significant (p = 0.53). A GEE model was used to evaluate the effect of HIV genotypes associated with changes of CD4+ T-cell count, using HIV genotype B as a reference group. There was no significant difference in rate of CD4+ cell count change between genotypes B, CRF01_AE, and recombinant B/CRF01_AE (p = 0.39). Similarly, no significant difference was observed in a comparison between infection with HIV genotype B and non-B genotype (p = 0.45), between CRF01_AE and non-CRF01_AE (p = 0.66), and between recombinant B/CRF01_AE and non-recombinant forms (p = 0.99). High PVL (>50,000 copies/mL) and high CD4+ T-cell counts (>500 cells/μL) at baseline were each significantly associated with more rapid CD4+ T-cell decline (p < 0.001). There was no association between age at diagnosis and rate of CD4+ T-cell decline (p = 0.19).

### HIV genotype and progression to immunologic failure

Among 189 HIV-infected MSM, 69 subjects reached criteria for immunological failure, i.e., either CD4+ T-cell level < 200 cells/μL (n = 34) or initiation of ART (n = 35). The proportion reaching immunologic failure did not significantly differ between those infected with CRF01_AE, HIV genotype B and B/CRF01_AE recombinants/dual infections (37%, 30% and 33% respectively, p = 0.852). Similarly, the proportion reaching failure was not significantly different between those aged 35 and over, and those younger than 35 (50% vs. 35%, p = 0.185). However, the proportion reaching immunologic failure was significantly greater in those with low baseline CD4+ T-cell counts (≤500 cells/μL) than those with high baseline CD4+ T-cell counts (>500 cells/μL, 45% vs. 24%, p = 0.003), and in those with high vs. low PVL (i.e., greater than vs. less than 50,000 copies/mL, 44% vs. 24%, p = 0.005).

In Kaplan-Meier analyses, there was no significant difference in survival probability from EDI to immunological failure by HIV genotype. However, baseline PVL > 50,000 copies/mL was associated with higher likelihood of failure; the unadjusted Cox proportional hazard ratio (HR) was 2.9 (95% confidence interval [CI] 1.6–5.1, p < 0.001) for those with baseline VL > 50,000 copies/mL compared with baseline PVL ≤ 50,000, and the HR remained significant after adjustment for baseline CD4+ T-cell and age (AHR 2.0, 95% CI 1.1–3.6, p = 0.017) (Table 2). Likewise, participants with initial CD4+ T-cell CD4 ≤ 500 cells/μL had a significantly greater likelihood of progression to clinical failure relative to participants with CD4>500 after adjustment for baseline PVL and age (AHR 2.0, 95% CI 1.1–3.4, p = 0.015) (Table 2).

**Table 2:** Hazard ratios (HRs) for clinical progression from estimate date of infection (EDI) to immunologic failure.

Variables	Number of immunologic failure* (n = 69)	Crude		Adjusted	
		HR (95% CI)	p value	HR (95% CI)	p value
HIV-1 subtype					
B	3	Reference		-	
CRF01_AE	59	1.34 (0.42–4.29)	0.62	-	
Recombinant B/CRF01_AE	7	1.16 (0.30–4.48)	0.83	-	
Age at HIV-1 conversion					
≤ 35 years	59	Reference		Reference	
> 35 years	10	1.88 (0.96–3.68)	0.07	1.75 (0.89–3.43)	0.11
Baseline CD4					
≤ 500 cells/μL	50	2.44 (1.43–4.14)	0.001	1.97 (1.14–3.40)	0.015
> 500 cells/μL	19	Reference			
Baseline viral load					
≤ 50000 copies/mL	16	Reference		Reference	
> 50000 copies/mL	53	2.87 (1.63–5.06)	<0.001	2.03 (1.14–3.63)	0.017

* Immunologic failure (n = 69) as defined by CD4 <200 cells/μL (n = 34) or initiation of ART (n = 35)

**Fig 1:**
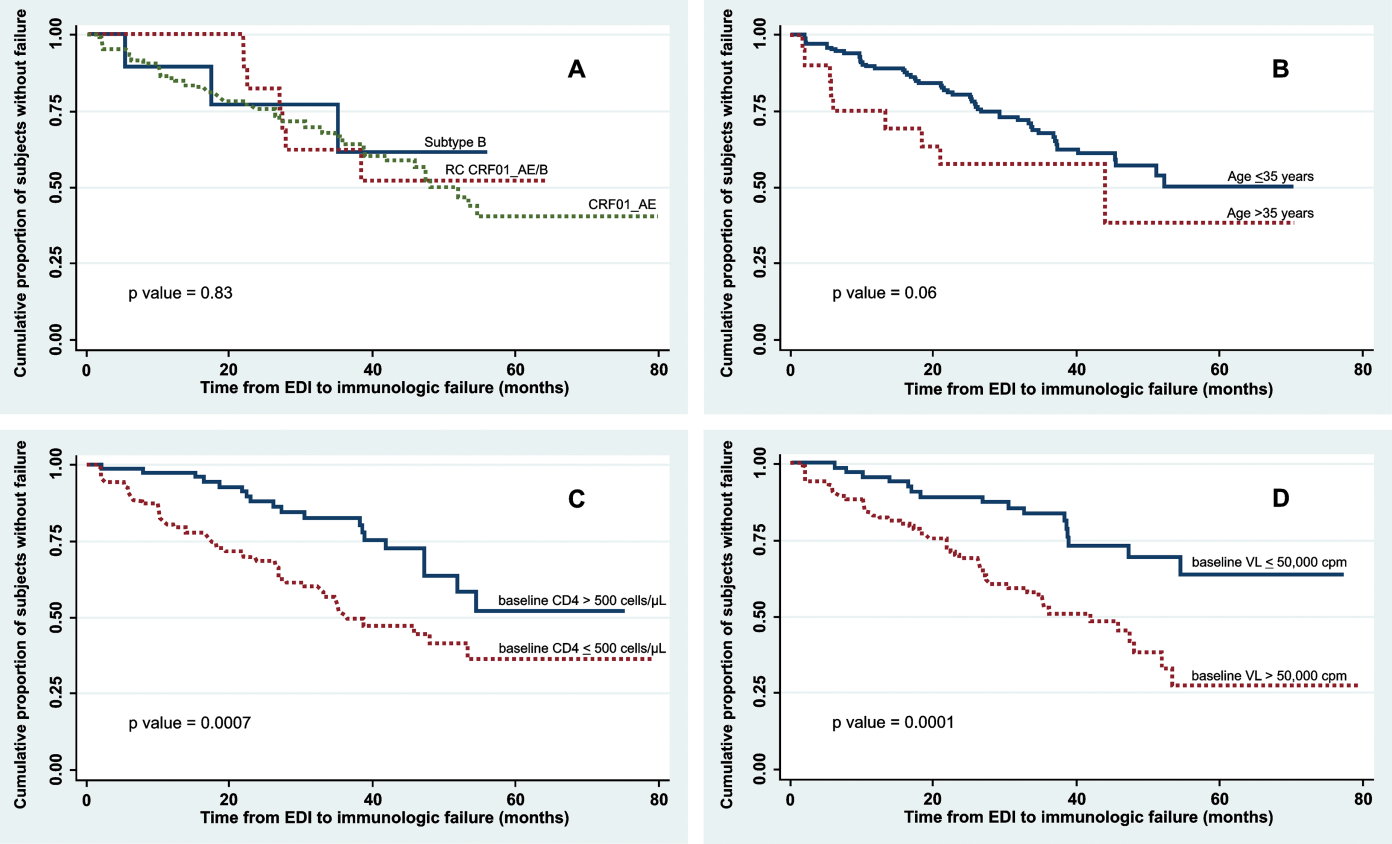
Kaplan-Meier survival curves. (A) Time to immunologic failure by infecting HIV subtype; (B) Time to immunologic failure by age at HIV conversion; (C) Time to immunologic failure by baseline CD4+ cell counts; (D) Time to immunologic failure by baseline viral load.

Among those with immunologic failure, the median time from EDI to failure was similar between MSM infected with CRF01_AE (18 months, IQR 8–33 months), genotype B (18 months, IQR 5–35 months) and recombinant B/CRF01_AE (27 months, IQR 22–28 months), p = 0.36. The median time from EDI to failure was 8 months (IQR 2–46 months) in older MSM and 22 months (IQR 2–55 months) in younger MSM, p = 0.06; 19 months (IQR 2–53 months) in MSM with high baseline PVL compared to 29 months (IQR 6–55 months) in low PVL, p = 0.0001; and 17 months (IQR 2–53) in MSM with low baseline CD4+ T-cell compared to 27 months (IQR 2–55) in high CD4+ T-cell count, p = 0.0007 (Fig 1).

## Discussion

We have examined factors associated with disease progression and immunologic failure in 189 newly HIV-infected Thai MSM in Bangkok, Thailand. Thai national HIV treatment guidelines evolved during the period between 2005, when ART was first included in universal health care coverage for all Thai citizens, and October 2014 when national standards adopted antiretroviral therapy for all HIV-infected patients regardless of CD4+ T cell count.[30–32] Observations recorded in this study were made between April 2006 and October 2014. Throughout the study, all BMCS participants received counseling and referral for ARV therapy at the time of diagnosis and at all follow-up visits. However, many MSM diagnosed with HIV infection nevertheless deferred initiation of therapy for a variety of reasons despite counseling and availability of counseling centers (see S1 File).

In this cohort, we found that high baseline CD4+ T-cell count and high baseline plasma viral load were independently associated with faster CD4+ T-cell decline, while the likelihood of immunologic failure was associated with low baseline CD4+ T-cell count and high baseline plasma viral load. However, infecting viral strain (i.e., CRF01_AE, HIV genotype B and inter-strain recombinants) did not significantly affect baseline CD4+ T-cell count, baseline plasma viral load, CD4+ T-cell decline rate, likelihood of reaching immunologic failure (CD4+ T-cell < 200 cells/μl or initiation of ART), or time to reach immunologic failure.

A substantial body of literature suggests that viral subtype may affect pathogenesis and disease progression during infection with HIV. This is most evident for HIV subtype D, which has been shown in multiple independent reports to be associated with more rapid CD4+ T-cell count decline and shorter time to AIDS than HIV subtypes A and B.[13–19] Research involving other subtypes has yielded less clear results, with some studies showing significant differences between subtypes,[16, 33] and others failing to do so.[25, 27, 34] However, three previous reports have associated CRF01_AE infection with unfavorable prognostic indicators when compared to other genotypes in co-circulation. Hu reported higher PVL (but no differences in CD4+ T-cell count) during infection with CRF01_AE compared with subtype B among PWID in Bangkok.[35] More recently, Ng reported faster CD4+ T-cell decline and shorter time to ART in CRF01_AE than other subtypes in circulation in Singapore,[33] and Li reported lower baseline CD4+ T-cell count, faster progression to AIDS and a greater frequency of CXCR4 tropism among MSM infected with CRF01_AE than those infected with subtype B and recombinants in Shanghai.[36, 37]

Directly comparing HIV subtypes is often complicated by factors such as uncertainty regarding mode of transmission and time of infection, diversity of host genetics, effect of comorbid conditions, small sample size, and the use of strain identification methods that do not reliably distinguish between subtypes or inter-strain recombinants. This analysis was restricted to a relatively homogeneous population of incident cases with a well-defined time of infection and known mode of transmission (sexual exposure), and we used robust methods to determine infecting viral strain. The overall CD4+ T-cell decline rate observed in our cohort (67 cells/μL/year) is comparable with previous reports ranging from 47–90 cells/μL/year[33, 38] and somewhat lower than that reported in the CASCADE study (130 cells/μL, for white men at 2 years post seroconversion).[26] Our results concur with other studies noting that high baseline PVL and low baseline CD4+ T-cell count are significantly associated with rapid clinical progression.[38–41] While at least two studies have reported a correlation between older age and accelerated CD4+ T-cell decline,[39, 41] we did not see this relationship in our study. In contrast with some previous reports, we did not observe differences in rate of disease progression in association with infecting viral strain.

Nevertheless, there are important limitations to this study. Participants becoming infected late during the period of enrollment/follow-up in our cohort study may have had only short post-infection follow-up time. The relatively small number of pure HIV genotype B infections may have limited our ability to detect modest differences between B and CRF01_AE and recombinant strains. The limited availability of sequence data in this analysis did not permit prediction of viral co-receptor tropism and hence the evolution of CXCR4 tropism as a marker of disease progression. Participant ART use was determined by history, and may not in all cases accurately reflect true ART use. We did not employ full-genome sequencing and it is possible that we failed to recognize recombination in some regions of the viral genome. This cohort population was a convenience sample of MSM who enrolled in the BMCS and may not represent findings among MSM in other locations in Bangkok or elsewhere in Thailand. Due to lack of regulatory approval, characterization of host genetic background was not undertaken in this study. Past studies have shown that certain HLA class I alleles B*35, Cw*04, A*30, B*45 are linked with accelerated disease progression,[42, 43] while alleles A*7401 and B*57 appears to be protective.[44, 45]

It is worth noting that despite the historical presence of subepidemics segregated by genotype and risk factor, CRF01_AE now appears to be rising to predominance in all risk groups throughout Asia.[22, 46–48] It remains to be determined whether this can be accounted for by genetic drift, or if there are inherent differences between strains such as baseline plasma viral load, inherent transmissibility or other biological or epidemiologic factors that may underlie this shift. Unfortunately, a significant number of MSM reached immunologic criteria for AIDS despite the existence of the Guidelines for antiretroviral therapy in HIV-1 infected adults and adolescents 2014, Thailand,[32] highlighting the ongoing need to address barriers to effective treatment, including fear of stigmatization and concern about the side effects of antiretroviral drug therapy.

## Supporting information

S1 File Background information on ethical review and access to antiretroviral treatment in the Bangkok Men Who Have Sex with Men Cohort Study. (DOCX)

S2 File Dataset used for statistical analyses in this study. (XLSX)
