# Supplementary material for: Association between HIV genotype, viral load and disease progression in a cohort of Thai men who have sex with men with estimated dates of HIV infection
Source: PLoS One. 2018 Jul 31;13(7):e0201386. doi: 10.1371/journal.pone.0201386 (PMC6067726; doi:10.1371/journal.pone.0201386)
Supplement: S1 File — (DOCX) [file pone.0201386.s001.docx]

**Background information on ethical review and access to antiretroviral treatment in the Bangkok Men Who Have Sex with Men Cohort Study**

**Contents**

1. Overview
2. Development and ethical oversight of the Bangkok MSM cohort study (BMCS)
3. Study participant access to information and antiretroviral therapy
   1. Role of the CDC in healthcare
   2. Universal health care coverage in Thailand
   3. Recommendations for antiretroviral treatment (ART) in Thailand
   4. Patient education and referral for ART at Silom Community Clinic (SCC) in BMCS
4. Ethical considerations and potential conflicts of interest
5. Citations

**1. Overview**

Because of the unique circumstances present at the Bangkok MSM cohort study site, and the evolution of HIV national healthcare policies in Thailand between 2006 and 2014, it is appropriate to provide some background information on these issues in this supplement resection. On behalf of all contributing authors, the corresponding author would be pleased to provide any additional information related to this publication upon request.

**2.** **Development and ethical oversight of the Bangkok MSM Cohort Study**

The analysis presented in the current manuscript is based on information obtained retrospectively from the Bangkok MSM cohort study (BMCS). The BMCS was a collaborative study undertaken by the US Centers for Disease Control and Prevention (CDC) and The Thai Ministry of Public Health (MOPH) with the primary objective of determining the prevalence and the incidence of HIV-1, HBV and sexually transmitted disease (STD) and characterizing HIV-1 among men who have sex with men (MSM) residing in Bangkok, Thailand. The study was initiated in 2005 after review by the US Centers for Disease Control Institutional Review Board (CDC IRB) and the Thai Ministry of Public Health Ethics Review Committee (MOPH ERC), and enrollment began in early 2006. During the course of the study, continuation reviews were performed annually, and special reviews were performed after each protocol modification by both the CDC and the Thai Ministry of Public health, until the conclusion of the study in 2017. The last continuation reviews occurred in July 2017 (CDC IRB) and February 2017 (MOPH ERC). We provide below a table of review of dates at each institution.

| **Protocol** | **Authority** | **Version** | **Submission date** | **Approval date** | **Expir. date** |
| --- | --- | --- | --- | --- | --- |
| Protocol V1 | CDC | BMCS4614_V1_IRB | 12-Jan-05 | N/A | N/A |
| Protocol V2 | CDC | BMCS4614_V2_IRB | 17-Jan-05 | N/A | N/A |
| Protocol V3 | CDC | BMCS4614_V3_IRB | 25-Apr-05 | N/A | N/A |
| Protocol V4 | CDC | BMCS4614_V4_IRB | 6-May-05 | N/A | N/A |
| Protocol V5 | CDC | BMCS4614_V5_IRB | 28-Jun-05 | 10-Jul-05 | 10-Jul-06 |
| Amendment 1 | CDC | BMCS4614_V9-AM1_IRB | 28-Mar-06 | 26-Apr-06 | 10-Jul-06 |
| Continuation 1 | CDC | BMCS4614_V11-CT1_IRB | 15-May-06 | 16-Jun-06 | 10-Jul-07 |
| Continuation 2 | CDC | BMCS4614_V13-CT2_IRB | 21-May-07 | 22-Jun-07 | 10-Jul-08 |
| Amendment 2 | CDC | BMCS4614_V15-AM2_IRB | 13-Nov-07 | 13-Dec-07 | 10-Jul-08 |
| Continuation 3 | CDC | BMCS4614_V17-CT3_IRB | 27-May-08 | 9-Jul-08 | 10-Jul-09 |
| Amendment 3 | CDC | BMCS4614_V19-AM3_IRB | 13-Mar-09 | 20-Apr-09 | 10-Jul-09 |
| Continuation 4 | CDC | BMCS4614_V19-CT4_IRB | 15-Jun-09 | 3-Sep-09 | 9-Jul-10 |
| Amendment 4 | CDC | BMCS4614_V20-AM4_IRB | 8-Oct-09 | 24-Nov-09 | 9-Jul-10 |
| Continuation 5 | CDC | BMCS4614_V20-CT5_IRB | 3-Jun-10 | 12-Jul-10 | 9-Jul-11 |
| Amendment 5 | CDC | BMCS4614_V21-AM5_IRB | 28-Mar-11 | 16-Jun-11 | 9-Jul-11 |
| Continuation 6 | CDC | BMCS4614_V21-CT6_IRB | 4-Jul-11 | 15-Jul-11 | 9-Jul-12 |
| Continuation 7 | CDC | BMCS4614_V6 Apr 2011 | 31-May-12 | 2-Jul-12 | 9-Jul-13 |
| Amendment 6 | CDC | BMCS4614_V7 Sep 2012 | 14-Sep-12 | 26-Sep-12 | 9-Jul-13 |
| Continuation 8 | CDC | BMCS4614_V7 Sep 2012 | 22-May-13 | 9-Jul-13 | 9-Jul-14 |
| Amendment 7 | CDC | BMCS4614_V8 Feb 2014 | 2-Dec-13 | 7-Feb-14 | 9-Jul-14 |
| Continuation 9 | CDC | BMCS4614_V8 Feb 2014 | 21-May-14 | 9-Jun-14 | 9-Jul-15 |
| Amendment 8 | CDC | BMCS4614_V9 Nov 2014 | 27-Dec-14 | 13-Jan-15 | 9-Jul-15 |
| Continuation 10 | CDC | BMCS4614_V9 Nov 2014 | 9-Jun-15 | 22-Jun-15 | 9-Jul-16 |
| Amendment 9 | CDC | BMCS4614_V10 Mar 2016 | 12-May-16 | 14-Jun-16 | 9-Jul-16 |
| Continuation 11 | CDC | BMCS4614_V10 Mar 2016 | 15-Jun-16 | 21-Jun-16 | 9-Jul-17 |
| Continuation 12 | CDC | BMCS4614_V11 May 2017 | 12-Jul-17 | 17-Jul-17 | 9-Jul-18 |
| Protocol V6 | MOPH | BMCS4614_V6_ERC | 24-Jun-05 | N/A | N/A |
| Protocol V7 | MOPH | BMCS4614_V7_ERC | 3-Oct-05 | N/A | N/A |
| Protocol V8 | MOPH | BMCS4614_V8_ERC | 29-Nov-05 | 29-Dec-05 | 31-Dec-09 |
| Amendment 1 | MOPH | BMCS4614_V10-AM1_ERC | 3-Apr-06 | 27-Dec-06 | N/A |
| Continuation 1 | MOPH | BMCS4614_V12-CT1_ERC | 22-Dec-06 | 27-Dec-06 | 31-Dec-07 |
| Continuation 2 | MOPH | BMCS4614_V14-CT2_ERC | 24-Oct-07 | 14-Nov-07 | 13-Nov-08 |
| Amendment 2 | MOPH | BMCS4614_V16-AM2_ERC | 3-Mar-08 | 26-Mar-08 | N/A |
| Continuation 3 | MOPH | BMCS4614_V18-CT3_ERC | 27-Oct-08 | 6-Dec-08 | 05-Nov-09 |
| Am. 3 & Cont. 4 | MOPH | BMCS4614_V19-AM3_ERC | 20-May-09 | 17-Jun-09 | 16-Jun-10 |
| Amendment 4 | MOPH | BMCS4614_V20-AM4_ERC | 9-Dec-09 | 27-Jan-10 | N/A |
| Continuation 5 | MOPH | BMCS4614_V20-CT5_ERC | 27-May-10 | 9-Jun-10 | 08-Jun-11 |
| Continuation 6 | MOPH | BMCS4614_V21-AM5_ERC | 13-May-11 | 25-May-11 | 24-May-12 |
| Amendment 5 | MOPH | BMCS4614_V21-AM5_ERC | 15-Jul-11 | 14-Sep-11 | N/A |
| Amendment 6 | MOPH | BMCS4614_V7 Sep 2012 | 26-Sep-12 | 24-Oct-12 | N/A |
| Continuation 7 | MOPH | BMCS4614_V7-AM5_ERC | 14-May-12 | 16-May-12 | 15-May-13 |
| Spec. Col. Cont. 1 | MOPH | BMCS4614_V7_ERC | 7-Jun-12 | 13-Jun-12 | 12-Jun-13 |
| Continuation 8 | MOPH | BMCS4614_V7 Sep 2012 | 23-Apr-13 | 8-May-13 | 07-May-14 |
| Spec. Col. Cont. 2 | MOPH | BMCS4614_V7_ERC | 30-Apr-13 | 22-May-13 | 21-May-14 |
| Amendment 7 | MOPH | BMCS4614_V8 Feb 2014 | 3-Mar-14 | 12-Mar-14 | 07-May-14 |
| Continuation 9 | MOPH | BMCS4614_V8 Feb 2014 | 22-Apr-14 | 23-Apr-14 | 22-Apr-15 |
| Spec. Col. Cont 3 | MOPH | BMCS4614_V8_ERC | 1-May-14 | 14-May-14 | 13-May-19 |
| Am 8 & Cont. 10 | MOPH | BMCS4614_V9 Nov 2014 | 9-Mar-15 | 26-Mar-15 | 25-Mar-16 |
| Continuation 11 | MOPH | BMCS4614_V9 Nov 2014 | 26-Feb-16 | 17-Mar-16 | 16-Mar-17 |
| Am 9 & Cont. 12 | MOPH | BMCS4614_V10 Jan 2017 | 09-Feb-17 | 16-Mar-17 | 15-Mar-18 |
|  |  |  |  |  |  |

**Table 1.** **BMCS regulatory review actions by the CDC Internal Review Board (CDC-IRB Protocol #4614) and the Thai Ministry of Public Health Ethics Review Committee (MOPH-ERC Ref No 73/2548)**.

**3. Study participant access to information and antiretroviral therapy**

**A. Role of the CDC in health care**

The US Centers for Disease Control and Prevention conducts epidemiological research and issues policy recommendations for disease prevention and control, environmental health, and health promotion and education activities designed to improve health. The CDC adheres to the highest standards for ethical conduct of research in all of its research activities, both domestically and internationally. However, the CDC is not a direct health care organization. As a US government organization, the CDC partners with equivalent host-nation institutions such as ministries of public health in conducting overseas research, and provides treatment recommendations in accordance with host nation policies and guidelines.

**B. Universal healthcare coverage in Thailand**

Universal healthcare coverage for all Thai citizens was enacted in 2001 and fully implemented in 2002. Under this system, Thai citizens are ensured for the costs of medical care through one of three programs (Civil Service Medical Benefit Scheme [CSMBS], Social Security Scheme [SSS], and the Universal Coverage Scheme [UC]). The CSMBS provides health care to government employees, dependents and retirees. The Social Security system is a compulsory insurance program providing coverage for employees of private businesses. The Universal Coverage Scheme is the residual insurer in Thailand, providing coverage to anyone who is not eligible for the remaining two programs. Patients who do not wish to participate in the Universal healthcare system for any reason may also seek medical care independently in the private sector on a fee for service basis. ^1, 2^

**C. Recommendations for antiretroviral treatment (ART) in Thailand**

Antiretroviral therapy is provided at no cost under the Universal healthcare system according to national guidelines. National guidelines for the treatment of HIV infection in Thailand evolved between 2005 and 2014; ART was included in Universal healthcare coverage in 2005^1^, and at that time, national recommendations called for initiation of ART for all persons with HIV infection and either an AIDS-defining illness, symptomatic infection or a CD4 count less than or equal to 200 cell/µL. In October 2012, the minimum CD4 threshold for ART was increased to less than or equal to 350 cell/µL, and in October 2014 recommendations were issued for ART in all patients regardless of CD4 count^3^ (table 2).

| **Year** | **Thai National Recommendations** |
| --- | --- |
| 2001 | Universal health care coverage for all Thai citizens enacted (30 baht scheme) |
| 2002 | Universal health coverage in Thailand fully implemented |
| 2005 | Universal health coverage including ART for HIV-infected patients in Thailand |
| 2008 | Start treatment in AIDS, symptomatic HIV infection or asymptomatic CD4 < 200 cells/µL |
| 2012 | Start treatment in AIDS, symptomatic HIV-infected or asymptomatic CD4 < 350 cells/µL |
| 2014 | Start treatment irrespective of CD4 level, focus on patients with CD4 < 500 cells/µL |

**Table 2.** **Timeline for implementation of universal health care coverage and adoption of recommendations for use of antiretroviral therapy (ART) in HIV-infected individuals between 2002 and 2014.**

The integration of ART into the universal health coverage in 2005 has substantially improved uptake of ART^1^. ART coverage has steadily increased and was reported to cover more than 80% of people living with HIV (PLHIV) in 2014^4^. However, most PLHIV entered into treatment late in the course of disease with a relatively low CD4 (median 111 cells/ µL in 2013) while 67% of newly HIV-infected subjects who initiated ART had CD4 <200 cells/ µL^4^. Multiple interrelated factors have hampered ART uptake and caused attrition across the care cascade, including^4^:

1. Social stigma around the issues of HIV prevention and treatment
2. Knowledge gap among persons at risk of HIV infection and persons living with HIV infection regarding core issues related to HIV prevention, infection status, risk of transmission, and treatment options.
3. Fear of medication side effects (e.g. lipoatrophy)
4. Inadequate linkage between diagnosis and treatment
5. Insufficient retention across the prevention and care continuum
6. Limited availability of Public health care facilities with capacity to provide ART, patient tracking, and support for regimen adherence.
7. Inadequate patient tracking and coordination between community health organizations and the traditional public health sector

**D. Patient education and referral for ART at Silom Community Clinic (SCC) in BMCS**

The BMCS sought to overcome barriers to HIV prevention and treatment services by providing an environment where HIV prevention and care are normalized, by providing direct education and counseling, by providing diagnostic testing, and by providing referral to centers with the capacity to provide ART. The BMCS provided counseling, education, testing and referral services to all visitors free of charge. In-person education and counseling was reinforced by a written informed consent process verifying comprehension of a standardized approved body of knowledge and a written copy provided to participants. With regard to referral for treatment, the BMCS informed consent included the following language:

**Informed Consent Language:**

If you have HIV we will give you counseling and refer you for treatment and care…… If you are at risk for HBV infection, we will offer you free HBV vaccine. This will protect you from getting HBV infection. This will require two additional visits. If you have TB we will refer you for treatment. If you do not wish to join this study, HIV and STI counseling, HBV vaccination and TB evaluation are available at public and private clinics and hospitals. We can provide a list of these to you.

During the study, when HIV infection was identified, clinic staff provided information on how and where study participants could access treatment, and this information and additional messaging on prevention of transmission was provided at follow-up visits after study entry. In the present study, 189 HIV-infected individuals were identified between December 2006 and October 2013. Of these, 34% opted to begin ART; among these, 50% received treatment from a registered selected health care facility through SSS and CSMBS, 32% were covered by UC, 11% received coverage through a clinical trial and 7% elected for self-payment. Among these individuals, the median lapse time between diagnosis and start of therapy was 593 days, the overall median CD4 count prior to therapy was 227 cells/µL_._ Of note, lapse time and last CD4 count substantially improved in 2012 (median lapse time 123 days, median CD4 count 292 cells/µL) when revised national ART guidelines were issued (Table 2). In the BMCS, among individuals declining to seek ART, reasons cited included a concern about the possibility of disclosure of the HIV status and resulting social stigma, and concern about the possibility of appearance altering treatment side effects such as lipodystrophy resulting impact on social interactions. For many individuals, the disadvantages of ART outweighed intangible health benefits during asymptomatic infection.

**4.** **Ethical considerations and potential conflicts of interest**

Unrecognized HIV infection, and high risk behavior leading to extremely high rates of new HIV infection in specific risk groups (MSM and particularly young MSM) are a major public health concern in Thailand. In fact, the annual incidence rate of HIV infection in MSM between the ages of 18 and 21 the Bangkok metropolitan area remains approximately 10%^5^, and onward transmission continues due to lack of recognition of infection, as well as inadequate knowledge of transmission risk factors, measures to prevent acquisition and onward transmission, available treatment options and negative perceptions about the risks of ART. Major objectives of the BMCS were to enhance awareness of the risk of HIV transmission in at–risk individuals, provide frequent testing and early recognition of infection status, provide counseling on risk behavior for at risk and HIV-infected participants, and to provide information on treatment options, potential side effects, and sources of care. These messages were reinforced at each follow-up visit during participation in the BMCS. Through these interventions the BMCS sought to directly address the factors responsible for propagation of the HIV epidemic and reduce the incidence of HIV infection in Thailand.

In the present study, the authors aimed to examine the association between infecting HIV subtype and markers of disease progression. This study was first conceived in 2014 when study staff became aware of the possible importance of HIV subtype to disease progression, and the availability of historical data to address this question. The study was therefore retrospective in design. However, analysis of these data had no impact on the services provided to visitors to the Silom Community Clinic (SCC) throughout the history of its operation, and most importantly, had no impact on patient education, HIV risk reduction counseling, or on referral to public or private hospitals and other clinical sites responsible for providing ART. Therefore conduct of this study did not pose any conflict of interest to clinic personnel in their care of visitors to the clinic.

**5. Citations**

1. Ingun N, Narkpaichit C, Boongerd P. Thailand Health Information System Improvement Through Universal Health Coverage Implementation. J of Thai Medical Informatic Association. 2015; 2: 137-147

2. Antos JR 2007. Health care financing in Thailand: modeling and sustainability. mission report to the world bank. World Bank, Washington, DC. <http://documents.worldbank.org/curated/en/2007/08/15622368/health-care-financing-thailand-modeling-sustainability>.

3. Thailand AIDS Response Progress Report 2014

<http://files.unaids.org/en/dataanalysis/knowyourresponse/countryprogressreports/2014countries/THA_narrative_report_2014.pdf>

4. Thailand AIDS Response Progress Report 2015

<http://www.unaids.org/sites/default/files/country/documents/THA_narrative_report_2015.pdf>

5. Van Griensven F, Thienkrua W, McNicholl JM, et al. Evidence of an Explosive Epidemic of HIV Infection in a Cohort of Men Who Have Sex With Men in Thailand. AIDS. 2013; 27 (5): 825-832
